# Flame‐Retardant Quasi‐Solid‐State Electrolytes From Self‐Assembled Azolate Hybrid Frameworks for Highly Safe Lithium Batteries

**DOI:** 10.1002/anie.1279221

**Published:** 2026-06-24

**Authors:** Shun Wang, Qimin Zhu, Yuanyuan Tian, Peiyu Cui, Qing Ji, Tian Xie, Hong Wang, Dong Zhou, Guoxiu Wang, Wenhuan Huang

**Affiliations:** ^1^ Key Laboratory of Chemical Additives for China National Light Industry College of Chemistry and Chemical Engineering Shaanxi University of Science and Technology Xi'an People's Republic of China; ^2^ College of Chemistry and Chemical Engineering Flexible Energy Storage and Interfacial Chemistry Key Laboratory of Shaanxi University Shaanxi University of Science and Technology Xi'an People's Republic of China; ^3^ Department of Mechanical Engineering Vehicle Energy and Safety Laboratory Ningbo University of Technology Ningbo People's Republic of China; ^4^ Shanghai Lingfang Energy Co. Ltd. Shanghai People's Republic of China; ^5^ Tsinghua Shenzhen International Graduate School, Tsinghua University Shenzhen China; ^6^ Centre For Clean Energy Technology School of Mathematical and Physical Science Faculty of Science University of Technology Sydney Sydney New South Wales Australia

**Keywords:** azolate hybrid frameworks (AHFs), flame‐retardant, Li^+^ migration, quasi‐solid‐state electrolyte, thermal runaway

## Abstract

Achieving quasi‐solid‐state electrolytes (QSSEs) that simultaneously deliver fast ion transport and intrinsic thermal safety remains a central challenge for lithium batteries, as improvements in ionic conductivity are often coupled with increased flammability and interfacial instability. Here, we present a spray‐assisted in situ assembly strategy to construct azolate hybrid frameworks (AHFs) directly on glass fiber substrates, followed by thermal polymerization to yield a chemically integrated QSSE. The heterocyclic AHF provides ordered lithium‐philic coordination sites and continuous ion‐transport pathways, enabling efficient Li^+^ migration while maintaining high thermal robustness. As a result, the resulting LiFePO_4_|FP10v‐GF|Li cell sustains stable cycling for over 500 cycles at 25°C and 100 cycles at 60°C. Notably, the framework architecture enables molecular level confinement of triethyl phosphate (TEP) as a flame‐retardant component, establishing a nitrogen phosphorus synergistic flame‐retardant mechanism without compromising electrochemical compatibility. Consequently, high‐loading Li||LiFePO_4_ cells exhibit stable cycling under practical conditions (E/C = 0.56 g Ah^−1^, N/p = 3.27) and successfully withstand accelerated rate calorimetry tests from 25°C to 300°C without thermal runaway. This work demonstrates how framework chemistry and molecular confinement can be synergistically integrated to decouple ionic conductivity from flammability, providing a general design principle for intrinsically safe, high‐energy quasi‐solid‐state lithium batteries.

## Graphical abstract

This work develops a spray‐assisted in situ assembly technique to construct AHF materials with nitrogen/oxygen‐regulated one‐dimensional channels on glass fiber, providing abundant active sites and enabling rapid transport for lithium ions. Subsequently, phosphate flame retardants are encapsulated via in situ polymerization, resulting in a thermally stable flame‐retardant electrolyte based on a nitrogen‐phosphorus synergistic flame‐retardant mechanism. This approach effectively suppresses combustion, stabilizes the electrode‐electrolyte interface, and offers a feasible pathway for constructing high‐safety, high‐energy‐density quasi‐solid‐state lithium batteries.

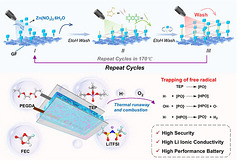

## 1 Introduction

**FIGURE 1:**
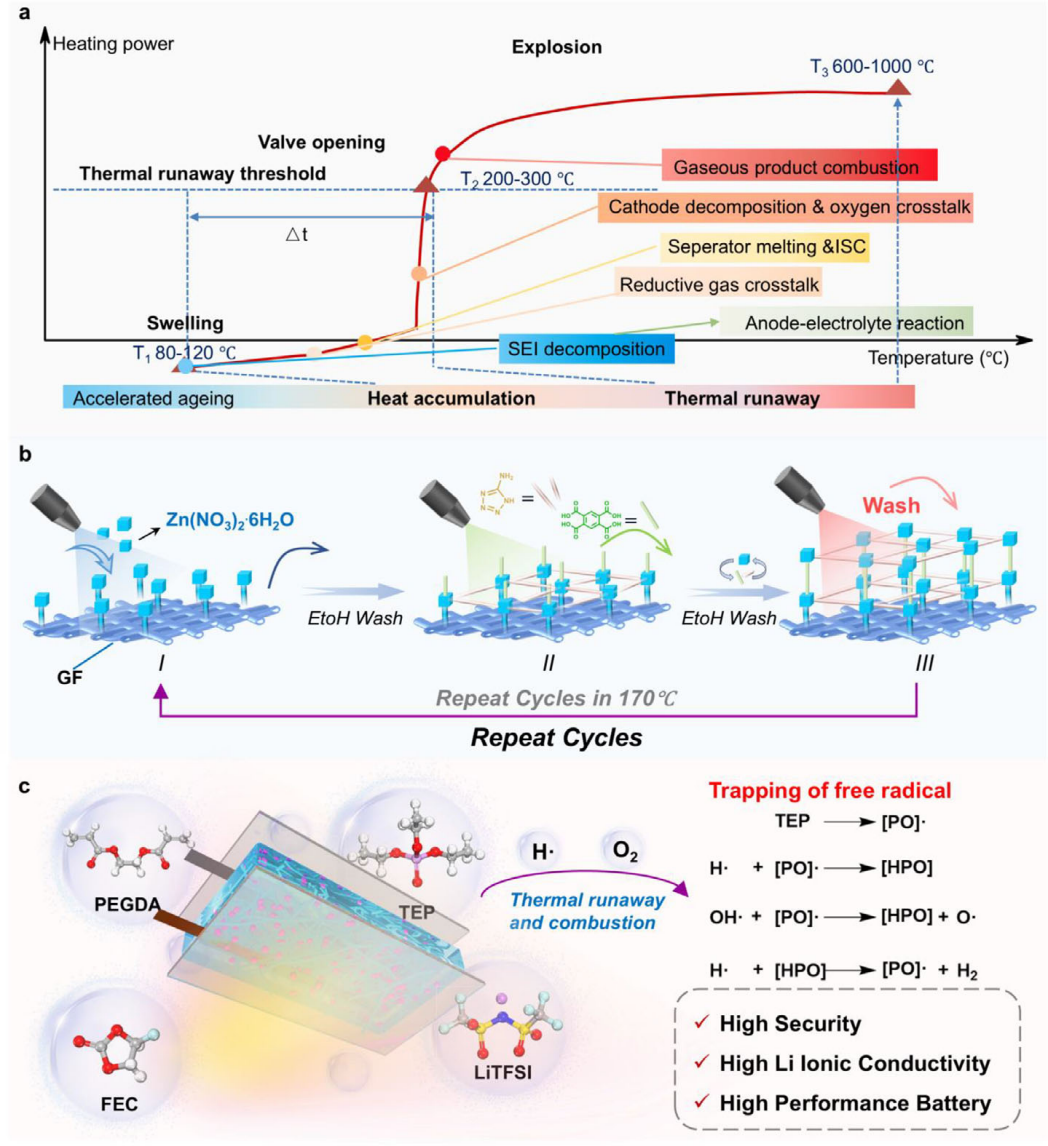
Overview of battery performance and thermal challenges. (a) Schematic illustration of the thermal runaway mechanism in conventional LIBs, showing onset points of key exothermic reactions at different stages. (b) In situ growth process of AHF‐PMA on glass fiber substrates. (c) Combustion mechanism diagram of quasi‐solid‐state batteries.

With the accelerating global energy transition, lithium‐ion batteries (LIBs) have become indispensable for electric vehicles and grid‐scale energy storage [1, 2, 3, 4]. However, the increasing prevalence of high‐temperature and extreme operating environments has exposed fundamental limitations in conventional battery technologies, making the development of thermally robust and intrinsically safe lithium batteries an urgent challenge [5, 6]. Although quasi‐solid‐state lithium batteries (QSSEs) are widely regarded as promising candidates due to their improved safety and high energy density, their stable operation under elevated temperatures remains far from resolved [7, 8]. Notably, no battery system can be considered absolutely safe, as thermal runaway is ultimately governed by internal heat accumulation arising from coupled electrochemical and chemical processes [9, 10]. Thermal runaway typically proceeds through accelerated aging, heat accumulation, and catastrophic failure, during which electrode degradation, interfacial collapse, and separator melting can induce internal short circuits and trigger highly exothermic reactions (Figure 1a) [11, 12, 13, 14, 15, 16]. These reactions, including electrolyte combustion, oxygen release from cathodes, and violent electrode oxidation, amplify heat generation and rapidly drive the system beyond controllable limits. Consequently, mitigating thermal runaway while preserving fast ion transport represents a central materials challenge for high‐safety solid‐state batteries, requiring electrolytes that simultaneously exhibit high ionic conductivity, thermal tolerance, and controlled chemical reactivity.

To address these challenges, extensive efforts have been devoted to optimizing QSSEs through composite structure design and functional integration [17, 18, 19]. Among various approaches, quasi‐solid‐state electrolytes based on metal‐organic frameworks (MOF‐QSSEs) have attracted considerable attention owing to their tunable porosity and capability to facilitate Li^+^ transport [20, 21, 22, 23]. Nevertheless, existing MOF‐QSSEs still suffer from intrinsic limitations. Excessive polymer plasticization and framework aggregation can compromise mechanical integrity and induce heterogeneous ion‐transport pathways [24, 25, 26, 27, 28]. Moreover, the organic nature of MOFs and residual liquid components renders these systems inherently flammable, posing significant safety risks under abusive thermal conditions [29, 30]. Although the incorporation of flame retardants into MOF‐based electrolytes appears attractive for improving thermal safety, such additives often disrupt lithium solvation structures and destabilize the lithium metal interface, resulting in deteriorated electrochemical performance [31, 32, 33]. Thus, the key unresolved challenge lies in how to chemically integrate flame‐retardant functionality with MOF‐based ion‐transport networks while maintaining interfacial stability and high ionic conductivity [34].

Herein, we address this challenge by developing a spray‐assisted in situ assembly strategy to construct an azolate hybrid framework (AHF‐PMA) directly on a glass fiber substrate (Figure 1b). The azolate hybrid framework provides abundant lithium‐philic N/O coordination sites and well‐defined ion‐transport channels, enabling regulated Li^+^ migration while preserving structural rigidity and thermal robustness. Coupled with in situ polymerization, this approach yields a QSSEs with high ionic conductivity (*σ_Li_^+^* = 1.436 × 10^−3^ S cm^−1^), a high Li^+^ transference number (*t_Li_^+^* = 0.63), and excellent electrochemical stability over a wide temperature range. As a result, lithium batteries based on this electrolyte exhibit long‐term cycling stability and good compatibility with high‐voltage LiNi_0.8_Co_0.1_Mn_0.1_0_2_ (NCM811) cathodes. More importantly, this framework architecture enables the molecular‐level confinement of triethyl phosphate (TEP) within the electrolyte via thermal curing, effectively transforming a conventional flame retardant into an integral, chemically regulated component of the ion‐conducting matrix (Figure 1c). The resulting nitrogen‐phosphorus synergistic flame‐retardant mechanism suppresses combustion and heat release, while the incorporation of fluoroethylene carbonate (FEC) mitigates interfacial side reactions between TEP and lithium metal. Through this integrated molecular and interfacial design, the electrolyte achieves intrinsic thermal safety without sacrificing electrochemical performance.

## 2 Results and Discussion

### 2.1 Framework‐Guided Construction of Quasi‐Solid‐State Electrolytes With Integrated Ion Transport and Thermal Robustness

We prepared a precursor solution by dissolving zinc nitrate hexahydrate (Zn (NO_3_)_2_·6H_2_O) in deionized water (H_2_O), which was then uniformly deposited on the surface of glass fiber surfaces via a spray‐coating process. Subsequently, a precursor solution containing 5‐aminotetrazole (5‐ATZ) and pyromellitic acid (PMA) was spray‐coated onto the modified glass fibers, followed by thermal treatment at 170°C to obtain the AHF‐PMA composite material (Figures S1 and S2). Meanwhile, AHF‐coated glass fiber membranes with different loading contents (denoted as 0v‐GF, 5v‐GF, 10v‐GF, and 20v‐GF) were prepared by controlling the volume of the sprayed precursor solution. The successful formation of AHF‐PMA on glass fiber (GF) was confirmed by x‐ray diffraction (XRD), where diffraction peaks of the powders, in situ grown 5v‐GF, and simulated patterns matched well (Figure S3). GF was selected for its self‐supporting capability and high liquid absorption (thickness ≈ 0.243 mm; Figure S4). Optical microscopy revealed uniform AHF distribution, with 10v‐GF exhibiting the most homogeneous morphology (Figures S5 and S6), while folding tests confirmed excellent mechanical flexibility. Thermogravimetric analysis (TGA) further demonstrated the high thermal stability of AHF‐PMA (Figure S7), ensuring reliable operation under elevated temperatures.

**FIGURE 2:**
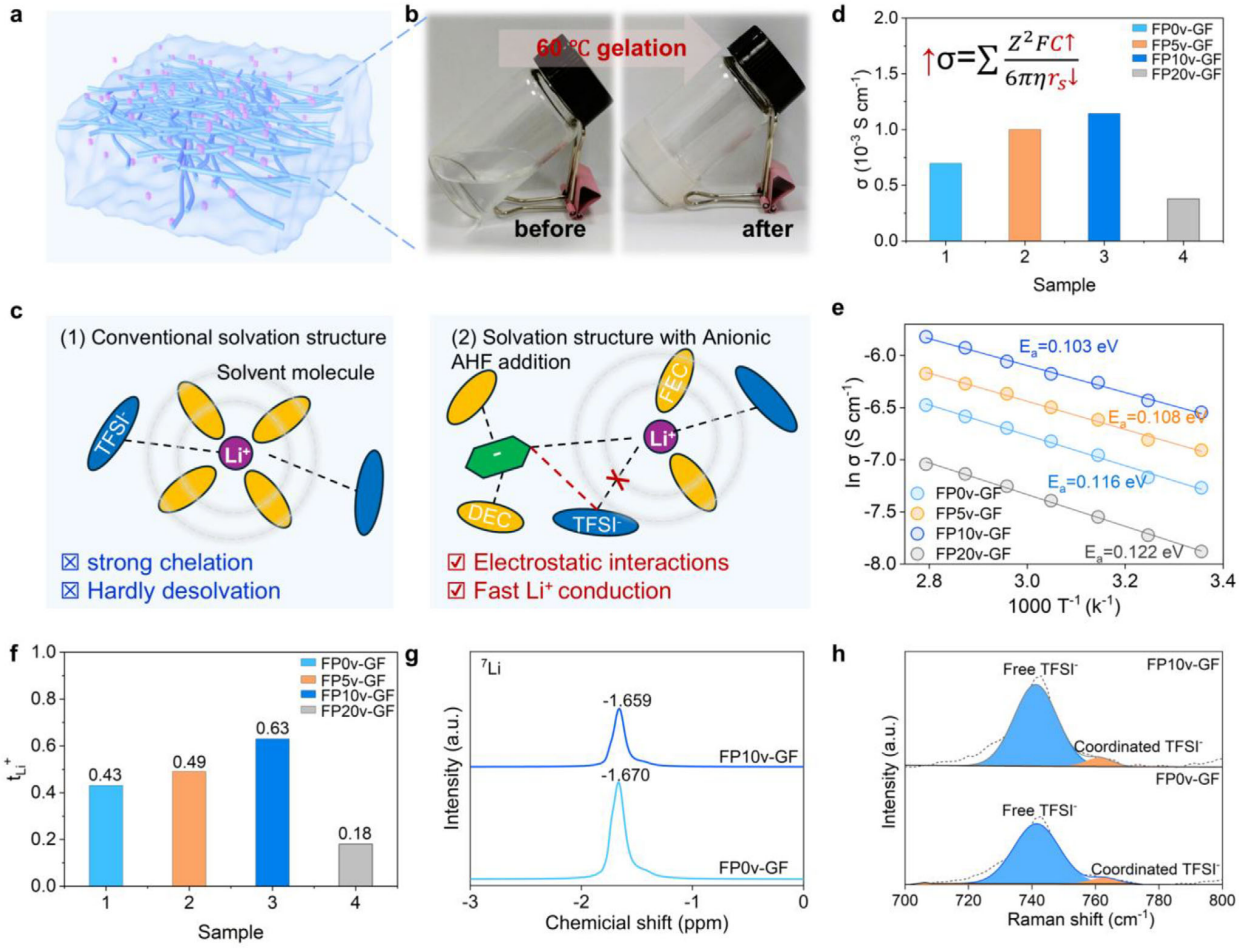
Structural and electrochemical characterization of FPxv‐GF (*x* = 0, 5,10, 20) solid‐state electrolytes. (a) Schematic diagram of the AHF‐PMA electrolyte (b) Optical images of the electrolyte before and after thermal polymerization. (c) Schematics of the Li‐ion solvation structure of Li ions in traditional electrolytes and the influence of AHF‐PMA in FPXV‐GF (*X* = 0, 5, 10, 20) electrolytes on the solvation structure of Li ions. (d, e) Ionic conductivities and activation energies of FPXV‐GF (*X* = 0, 5, 10, 20) electrolytes. (f) Comparison of the *t_Li_^+^* transference number of different relevant electrolytes. (g) Solid‐state ^7^Li NMR spectra of FP0v‐GF and FP10v‐GF. (h) Raman spectra of FP0v‐GF and FP10v‐GF.

The polymerizable precursor solution composed of polyethylene glycol diacrylate (PEGDA) and 2, 2'‐azobis (2‐methylpropionitrile) as a thermal initiator dissolved in liquid electrolyte (LE; 1 M Lithium bis(trifluoromethanesulfonyl)imide (LiTFSI) in diethyl carbonate (DEC)/fluoroethylene carbonate (FEC) [1:1, v/v]), was injected into the Xv‐GF (*X* = 0, 5, 10, and 20) membranes. The Xv‐GF (*X* = 0, 5, 10, 20) membranes filled with the precursor solution were assembled in a cell, which was placed at 80°C for in situ thermal polymerization to prepare corresponding solid polymer electrolytes (FPXv‐GF [*X* = 0, 5, 10, 20]) (Figure 2a,b, Figure S8). The thickness of the post‐assembly membrane is reduced to approximately 117 µm, which represents a decrease of about 51.9% compared to the original glass fiber membrane (∼243 µm, Figure S9). This reduction is primarily attributed to the filling of interfiber voids by the polymer binder and the compaction of the fiber network during the curing process. Such densification not only significantly enhances the mechanical strength and structural uniformity of the membrane but may also correspondingly alter its porosity and electrolyte affinity. Besides, as shown in Figure 2b, the LE system containing initiator azobisisobutyronitrile (AIBN) can form a leakage free solid‐state transparent gel material through thermal polymerization of PEGDA, demonstrating excellent anti‐leakage properties. SEM and elemental mapping analyses confirmed that the polymerized electrolyte membrane possesses a smooth surface morphology with homogeneous distribution of Zn, C, N, O and F elements (Figures S10–S21).

Further analysis of the solvent structures in the traditional electrolyte and the FPXv‐GF (*X* = 0, 5, 10, 20) systems (Figure 2c) reveals that conventional Li‐ion battery electrolytes typically adopt a “solvent‐separated ion pair” (SSIP) configuration. This structure imposes a high energy barrier to Li‐ion desolvation at the electrode interface, thereby limiting transport kinetics. Through analysis of the charge distribution in AHF‐PMA and its interactions with Li ions, this study finds that negative charges are predominantly localized at the N and O sites of the AHF‐PMA framework (Figures S22 and S23). Additionally, AHF‐PMA exhibits strong binding energy with both Li^+^ and bis(trifluoromethanesulfonyl)imide (TFSI^−^) anions. These results indicate that the carboxyl oxygen and tetrazole groups in AHF‐PMA can coordinate with Li^+^, reducing the solvent sheath structure (Stokes radius of solvated Li^+^, *rs*), lowering the desolvation energy barrier, and promoting Li‐ion release. Furthermore, the 5‐ATZ cations in the AHF‐PMA framework suppress anion migration through strong electrostatic interactions, thereby reducing the dissociation energy barrier of the Li salt and enhancing the Li‐ion concentration.

### 2.2 Regulated Li^+^ Transport and Electrochemical Stability by Azolate Hybrid Frameworks

In QSSEs, ionic conductivity (*σ*) is a decisive factor governing ion transport efficiency and serves as a critical metric for designing high‐performance electrolyte materials [35, 36]. To investigate the electrochemical properties of the FPXv‐GF (*X* = 0, 5, 10, 20) electrolytes, electrochemical impedance spectroscopy (EIS) tests of the symmetric cells were performed to evaluate the Li^+^ conductivity. Temperature‐dependent ionic conductivity measurements (Figure 2d, Figures S24 and S25, and Table S1) show that the symmetrical topology enables FP10v‐GF to maintain superior conductivity across the entire temperature range. And the room‐temperature ionic conductivity of FP10v‐GF is 1.44 × 10^−3^ S cm^−1^, superior to that of FP0v‐GF (*σ_Li_^+^* = 6.97 × 10^−4^ S cm^−1^), FP5v‐GF (*σ_Li_^+^* = 1.0 × 10^−3^ S cm^−1^) and FP20v‐GF (*σ_Li_^+^* = 3.78 × 10^−4^ S cm^−1^). Arrhenius analysis (Figure 2e) confirms a lower activation energy (*Ea* = 0.103 eV) for FP10v‐GF compared to FP0v‐GF (*Ea* = 0.116 eV), FP5v‐GF (*Ea* = 0.108 eV), and FP20v‐GF (*Ea* = 0.122 eV), indicating more favorable transport energy barriers. Besides, due to the presence of AHF‐PMA reducing the solvation energy around Li ions, decreasing the solvation sheath structure (*rs*), and increasing the Li^+^ concentration (*C*), Equation 3 further confirms the enhanced Li^+^ transport capability by AHF. Considering the concurrent migration of both cations and anions in solid electrolytes, the Li^+^ transference number (*t_Li_^+^*) was measured to evaluate the efficiency of Li‐ions transport. As shown in Figure 2f (Figures S26 and S27 and Table S2), FP0v‐GF and FP5v‐GF exhibited identical *t_Li_^+^* values of 0.49, while FP20v‐GF showed a significantly lower value of 0.18. In striking contrast, FP10v‐GF reached a remarkable *t_Li_^+^* of 0.63, demonstrating its effectiveness in suppressing TFSI^−^ mobility while enhancing Li^+^ conduction. Overall, FP10v‐GF electrolyte demonstrates superior electrochemical performance in both ionic conductivity and Li^+^ transference number compared to state‐of‐the‐art electrolytes (Table S3). Tafel analysis of Li||Li cells (Figure S28) further indicated an enhanced exchange current density (*I_0_* = 0.11 mA cm^−2^) for FP10v‐GF, demonstrating accelerated Li^+^ diffusion and uniform transport that mitigates dendrite growth. This result indicates that the incorporation of AHF‐PMA material effectively enhances the interfacial stability of the electrolyte system.

To investigate the chemical environment and mechanisms of Li^+^ transport in different electrolytes, ^7^Li solid‐state nuclear magnetic resonance (NMR) and Raman spectra measurements were performed on various electrolytes to understand the enhanced Li^+^ transport in FP10v‐GF and FP0v‐GF. As shown in Figure 2g, the downfield shift of the Li^+^ peak in FP10v‐GF signifies a significantly increased proportion of free Li^+^ ions. Furthermore, Raman spectroscopy directly quantified free TFSI^−^ and ion cluster concentrations (Figure 2h), revealing the electrolytes' Li^+^ dissociation efficiency. As shown in Figure 2h, the FP10v‐GF electrolyte exhibited a higher concentration of free TFSI^−^ (94.53%) and a correspondingly lower concentration of ion clusters (LiTFSI) (5.47%) compared to FP0v‐GF (93.49% free TFSI^−^). Therefore, the porous and topological framework of the AHF‐PMA, uniformly functionalized with lithiophilic N/O sites, facilitates homogeneous Li^+^ deposition.

### 2.3 Battery Performance of FPXv‐GF (*X* = 0, 5, 10, 20) Quasi‐Solid‐State Li Metal Batteries

**FIGURE 3:**
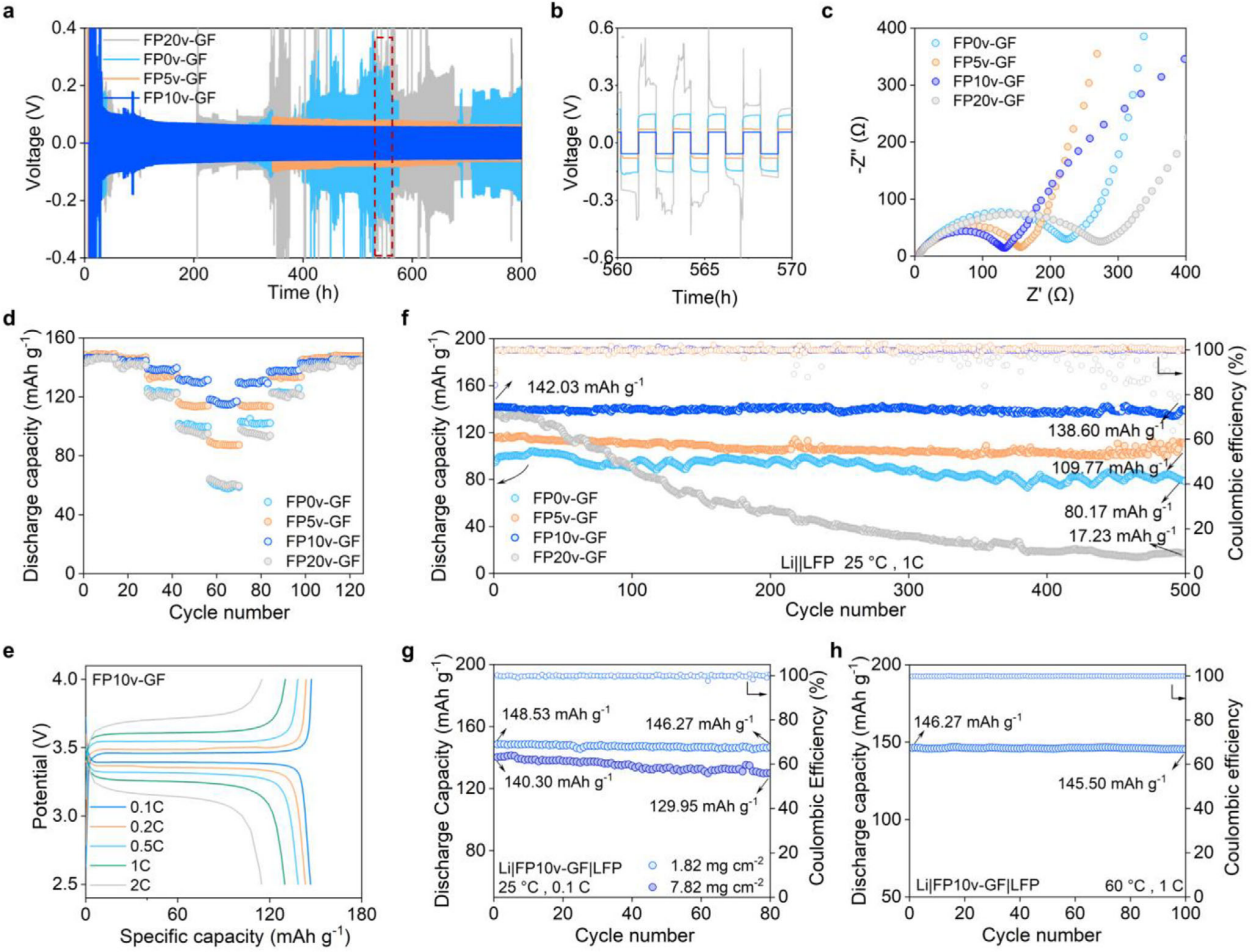
Electrochemical performance of FPXv‐GF (*X* = 0, 5, 10, 20) quasi‐solid‐state batteries. (a, b) Galvanostatic cycling curves of Li||Li cells using FPXv‐GF (*X* = 0, 5, 10, 20) at a current density of 1 mA cm^−2^. (c) Electrochemical impedance spectroscopy (EIS) of FPXv‐GF (*X* = 0, 5, 10, 20) membranes assembled in symmetric Li||LFP cells. (d, e) Rate performance and corresponding charge‐discharge curves for LFP|FPXv‐GF (*X* = 0, 5, 10, 20)|Li cells at different rates. (f) Cycling performance of LFP|FPXv‐GF (*X* = 0, 5, 10, 20) |Li cells at 1 C. (g) Cycling performance of high‐loading LFP|FP10v‐GF|Li cells at 0.1 C. (h) Cycling stability of the LFP|FP10v‐GF|Li cell at 60°C.

To elucidate the regulatory mechanism of AHF‐PMA topological structure on lithium‐metal interfacial stability, Li||Li symmetric cells employing FPXv‐GF (*X* = 0, 5, 10, 20) electrolytes were systematically evaluated at a current density of 1 mA cm^−2^. As illustrated in Figure 3a,b, the Li|FP10v‐GF|Li symmetric cell exhibits exceptional cycling stability for 800 h at 1 mA cm^−2^ with 1 mAh cm^−2^ capacity, maintaining a consistently lower overpotential (60 mV) compared to conventional Li|FPXv‐GF (*X* = 0, 5, 20)|Li cells. Moreover, the FP10v‐GF electrolyte demonstrated highly stable electrochemical performance over a wide range of current densities, from 0.2 to 5.0 mA cm^−2^ (Figure S29). Furthermore, XRD analysis of the FP10v‐GF membrane shows that after 100 h of cycling in a Li||Li battery, the characteristic diffraction peak at 6.77° remains present (Figure S30). This indicates that the material maintains structural stability during cycling without significant collapse, thereby providing crucial support for the subsequent high performance and long‐term cycling stability of the battery.

Notably, Li|FP10v‐GF|Cu yielded a higher CE of 91.7% than that of the Li|FP0v‐GF|Cu (82.5%) (Figure S31). This can be attributed to the superior Li^+^ transfer kinetics of FP10v‐GF, which effectively suppresses parasitic reactions. Furthermore, linear sweeping voltammetry (LSV) tests were conducted to assess the electrochemical stabilities of the FPXv‐GF (*X* = 0, 5, 10, 20) electrolytes (Figure S32). The incorporation of AHF‐PMA into the electrolytes enhances the oxidation potential to exceed 4.5 V, thereby demonstrating the suitability of the FPXv‐GF (*X* = 0, 5, 10, 20) electrolytes for high‐voltage positive electrodes. Furthermore, DFT calculations are conducted to evaluate the high‐voltage stability of electrolytes (Figure S33). As shown in Figure S33, the AHF‐PMA exhibits the HOMO energy level of −0.237 eV, indicating strong oxidative stability. Meanwhile, AHF‐PMA exhibits the LUMO energy level of −0.148 eV, indicating strong reduction stability. These results further support our hypothesis that the AHF structure can enhance the oxidation stability of the electrolyte, thereby contributing to the overall improvement of battery interfacial stability.

To elucidate the influence of the AHF‐PMA topological structure design on SEI composition, X‐ray photoelectron spectroscopy (XPS) analysis was performed on cycled lithium‐metal anodes (500 h, Li||Li symmetric cells). Compared to the FP0v‐GF system, the lithium metal interface with FP10v‐GF shows a substantial increase of lithium fluoride (LiF, 685.5 eV) and lithium nitride (Li_3_N,395.5 eV) content in the SEI layer, consistent with the results shown in Figure S34 and Figure S35. Notably, the hybrid SEI enriched with inorganic LiF and Li_3_N components contributes to enhanced stability at the solid electrolyte/anode interface. In particular, the high Young's modulus and high surface energy of LiF help homogenize the Li‐ion flux, thereby providing a reliable foundation for the cycling stability of the battery.

Further investigations into the charge transfer resistance (*R_ct_*) at the electrode‐electrolyte interfaces before rate testing were conducted to elucidate the Li^+^ transport dynamics at the interface (Figure 3c). The LiFePO_4_ (LFP)|FP10v‐GF|Li cell exhibited the lowest *R_ct_* of 132 Ω. In contrast, the LFP|FP0v‐GF|Li, LFP|FP5v‐GF|Li, and LFP|FP20v‐GF|Li systems showed significantly higher *R_ct_* values of 223.4, 155.5, and 277.7 Ω, respectively. Besides, the CV results (Figure S36) show that the Li|FP10v‐GF|LFP system has smaller peak potential differences and higher peak current densities, suggesting that it exhibits faster reaction kinetics and lower polarization. These results indicate that systems based on FP0v‐GF, FP5v‐GF, and FP20v‐GF possess substantially larger charge transfer resistances. Notably, the LFP|FP10v‐GF|Li configuration demonstrates superior electrode‐electrolyte interfacial contact and a higher potential to facilitate more uniform Li^+^ deposition, thereby contributing to the overall performance enhancement observed in cell applications.

After recognizing the superior stability and performance of symmetric Li cells, the application potential of FP10v‐GF in QSSLMBs was further evaluated by systematically investigating solid‐state electrolytes assembled with LiFePO_4_ (LFP) and LiNi_0.8_Mn_0.1_Co_0.1_O_2_ (NCM811) cathodes. The rate capability of the Li|FPXv‐GF (X = 0, 5, 10, 20)|LFP cells was systematically evaluated across current densities ranging from 0.1 C to 2 C (Figure 3d,e, Figure S37). At low current densities of 0.1 and 0.2 C, the cells assembled with FPXv‐GF (*X* = 0, 5, 10, 20) exhibit comparable specific capacities, owing to less stringent requirements of Li^+^ transport. At a current density of 2 C, the cell assembled with FP10v‐GF has a specific capacity of 118.2 mAh g^−1^, outperforming those with FP0v‐GF (64.6 mAh g^−1^), FP5v‐GF (89.5 mAh g^−1^) and FP20v‐GF (64.18 mAh g^−1^). This performance enhancement is attributed to the formation of a stable interface and superior ionic dynamics.

Furthermore, the long‐term cycle stability of the Li|FPXv‐GF (*X* = 0, 5, 10, 20)|LFP has been evaluated at a current density of 1 C (Figure 3f). It was found that the obtained Li|FP10v‐GF|LFP (negative/positive ratio, N/P = 19.68, electrolyte weight to cathode capacity ratio, E/C = 0.224 g Ah^−1^) not only presents good rate capability, but also displays a stable cyclability of 500 cycles at 1 C with a higher capacity retention of 97.59%. Meanwhile, the Li|FP10v‐GF|LFP cell demonstrates excellent cycling stability at rates of 0.1 C and 2 C, even under high‐loading LiFePO_4_ cathode (0.1 C, 7.82 mg cm^−2^, Li||LFP cell, N/P = 3.27, E/C = 0.056 g Ah^−1^) conditions (Figure 3g, Figure S38). In addition to the LFP cathode, the compatibility of FP10v‐GF with a high‐voltage Ni‐rich cathode, NCM811, was also investigated. The Li|FP10v‐GF|NCM811 cell demonstrated a high coulombic efficiency of 97.5% and retained excellent specific capacity at both 0.1 C and 1 C rates under high‐voltage operating conditions (Figures S39 and S40). Moreover, previous linear sweep voltammetry (LSV) tests (Figure S32) revealed that the FP10v‐GF electrolyte exhibits a broad electrochemical stability window of 5.05 V, demonstrating its compatibility with high‐voltage NCM811 cathodes and ensuring stable battery operation. This superiority becomes more pronounced under elevated temperatures (Figure 3h). Notably, the FP10v‐GF cell achieves 99.47% capacity retention after 100 cycles at 1.0 C and 60°C. We therefore speculate that its excellent thermal stability and high specific capacity primarily originate from the intrinsic thermal stability of the AHF‐PMA material and the highly efficient, rapid ion transport channels within its topological structure. Overall, FP10v‐GF outperforms state‐of‐the‐art electrolytes across critical metrics—including capacity, rate capability, operating temperature range, and cycle life—endowing Li‐metal batteries with marked advantages. Its balanced performance is further quantified in Table S4.

### 2.4 Nitrogen‐Phosphorus Synergistic Flame Retardancy Enabled by Framework‐Constrained Phosphate Species

To further enhance the safety of batteries under high‐temperature conditions, we introduced the flame retardant triethyl phosphate (TEP) into the FP10v‐GF electrolyte to fabricate a flame‐retardant electrolyte membrane (denoted as TFP10v‐GF, with FP10v‐GF as the control). The scanning electron microscopy (SEM) and energy‐dispersive x‐ray spectroscopy (EDS) directly display the uniform distribution of the elements C, O, N, Zn, and P. As shown in Figure S41, the EDS mapping results demonstrate a homogeneous distribution of P elements throughout the membrane.

In order to explore the influence of TEP addition on the electrochemical properties of PEGDA electrolyte, FP10v‐GF and TFP10v‐GF membranes were prepared for comparison. The ionic conductivities of the prepared TFP10v‐GF samples were measured at different temperatures ranging from 25°C to 85°C using electrochemical impedance spectroscopy (EIS) (Figure S42), with the ionic conductivity and ionic activation energy (*σ_Li_^+^* = 1.356 × 10^−3^ S cm^−1^, *Ea* = 0.105 eV) obtained at room temperature for TFP10v‐GF. Compared with the FP10v‐GF electrolyte (*σ_Li_^+^* = 1.436 × 10^−3^ S cm^−1^, *Ea* = 0.103 eV), the conductivity of this electrolyte has decreased. This is mainly because TEP, as a high‐viscosity solvent, contains highly polar phosphate ester groups in its molecules, which significantly increase the overall viscosity of the electrolyte, thereby increasing the migration resistance of Li ions in the medium and ultimately leading to a reduction in ionic conductivity.

Furthermore, as shown in Figure S43, the Li ion transference number (*t_Li_^+^*) measured at 25°C is 0.72, which represents a significant improvement compared to FP10v‐GF (*t_Li_^+^* = 0.63). Meanwhile, frontier molecular orbital energy calculations (Figure S44) indicate that TEP possesses a lower HOMO energy level (−0.25 eV) and a higher LUMO energy level (0.016 eV). This suggests that it can enhance the oxidation resistance stability of the electrolyte and facilitate the reduction of TFSI^−^ and the formation of LiF, thereby effectively reducing the proportion of organic components in the SEI layer. The aforementioned performance improvements are primarily attributed to the strong electron‐donating ability of the P═O group in TEP molecules. This group forms strong Lewis acid‐base interactions with the TFSI‐ anion in the Li salt, effectively restricting anion migration, slowing down their movement, relatively increasing the cation concentration in the system, and ultimately significantly enhancing the Li ions transference number.

To investigate the influence of TEP on the electrode/electrolyte interface and its preferential adsorption behavior, we disassembled the Li|TFP10v‐GF|LFP cell after 10 cycles and performed XPS analysis on both the separator and Li metal interfaces (Figure S45). The results indicate the presence of Li_3_PO_4_ and LiPO_2_F_2_ at both interfaces, with a stronger LiPO_2_F_2_ signal observed at the separator interface. This benefit stems from TEP's optimization of the Li‐ion solvation structure, which promotes the reaction between TFSI^−^ anions and FEC. Simultaneously, when coexisting with carbonate co‐solvents such as FEC, TEP mitigates its inherent corrosiveness toward Li metal, thereby facilitating the formation of a stable SEI layer and protecting the Li metal interface. Additionally, the porous structure and affinity of the glass fiber separator facilitate the infiltration and retention of TEP. These findings suggest that TEP preferentially adsorbs at the separator interface while also contributing to the stabilization of the Li metal interface, thereby providing support for the long‐term cycling stability of the battery.

**FIGURE 4:**
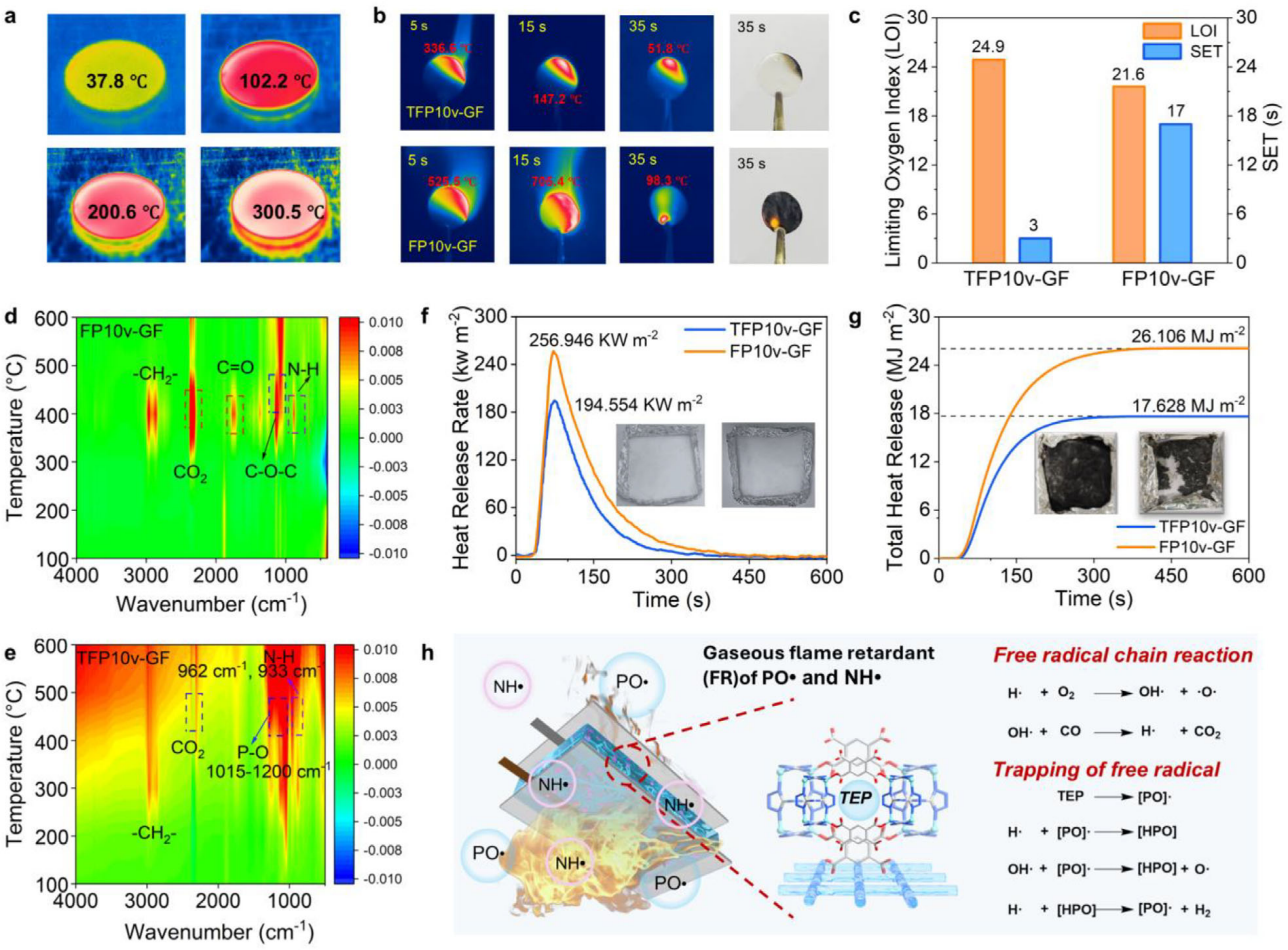
Nitrogen‐phosphorus synergistic flame‐retardant characterization of TFP10v‐GF. (a, b) Infrared thermal images and ignition experiments of TFP10v‐GF and FP10v‐GF membranes from 25°C to 300°C. (c) Limiting oxygen index (LOI) and self‐extinguishing time (SET) histograms of TFP10v‐GF and FP10v‐GF membranes. (d, e) The TGA‐FTIR of FP10v‐GFand TFP10v‐GF membranes. (f, g) The micro‐combustion calorimeter (MCC) test of TFP10v‐GF and FP10v‐GF membranes from 500°C: (f) Heat release rate (HRR) curves, (g) Total heat release (THR) curves and (h) the flame‐retardant mechanism of TFP10v‐GF membrane.

To evaluate the effect of adding TEP on the flame‐retardant properties of the electrolyte. Thermal shrinkage and combustion tests were carried out to simulate the thermal performance of TFP10v‐GF and FP10v‐GF under different heat‐abused conditions. As shown in Figure 4a, the FP10v‐GF membrane exhibited no significant shrinkage during heating from room temperature to 300°C. Its excellent thermal stability significantly reduces the risk of thermal runaway caused by internal battery short circuits. To evaluate the safety of the designed electrolyte, combustion tests and self‐extinguishing tests (SET) were conducted using an infrared thermal imager. As shown in Figure 4b,c (Figure S46), the TFP10v‐GF electrolyte could not be ignited after the first 5 s of ignition and exhibited rapid self‐extinguishing behavior upon the second ignition attempt, with an SET time of only 3 s, demonstrating excellent flame retardancy. Besides, the LOI of TFP10v‐GF (24.9) was higher than that of FP10v‐GF (21.6). In flame retardancy classification, materials with an LOI below 21 can sustain combustion in air. If the LOI is between 21 and 28, the material is considered flame‐resistant [37, 38, 39, 40], as it is likely to burn in air and may self‐extinguish upon flame removal, consistent with our experimental results. This performance is attributed to the PO· radicals generated by the decomposition of phosphate groups in TEP, which combine with H• and OH• radicals, effectively blocking the free radical chain reaction pathway. Notably, infrared thermal imaging observations of the combustion processes of both electrolytes revealed that the surface temperature of TFP10v‐GF was significantly lower than that of FP10v‐GF. This phenomenon indicates that incorporating TEP not only significantly enhances the material's flame retardancy but may also improve its thermal conductivity. Meanwhile, combustion optical images show that both electrolytes form a dense carbon layer through pyrolysis during combustion (Figure 4b, Figure S47, Video S2 and Video S3). However, the pure glass fiber film burns instantly upon contact with flame (Video S1). This carbon layer acts as an effective barrier, isolating heat and oxygen, thereby suppressing further internal combustion and significantly improving the safety performance of the battery. In contrast, the SET time of FP10v‐GF without TEP was 16 s. Although its flame retardancy is inferior to that of TFP10v‐GF, the carbon layer formed during combustion can still partially isolate heat and oxygen, which is one of the reasons why the FP10v‐GF electrolyte can maintain stable operation at 60°C.

The thermal behavior of the electrolytes was further characterized using in situ thermogravimetry‐Fourier transform infrared spectroscopy (TGA‐FTIR). As shown in Figure 4d,e (Figure S48), TGA‐FTIR analysis reveals the gas‐phase flame‐retardant mechanism of the TFP10v‐GF system under thermal runaway conditions. The characteristic broad absorption peak in the range of 1115–1200 cm^−1^ directly confirms the thermal decomposition of the plasticizer TEP and its conversion into highly active phosphorus‐containing radicals such as PO•. Meanwhile, the characteristic double peaks at 933 and 962 cm^−1^ are clearly attributed to the pyrolysis products of the AHF‐PMA material skeleton, indicating its successful release of nitrogen‐containing active species as a nitrogen source. The above results demonstrate that, upon thermal triggering, the electrolyte system activates a well‐defined gas‐phase phosphorus‑nitrogen synergistic flame‐retardant pathway: TEP provides phosphorus‐centered active species (PO•) capable of quenching key radicals such as H• and OH•, while AHF‐PMA supplies nitrogen‐containing gas‐phase products that act synergistically. Together, they construct an efficient radical‑quenching network in the gas phase, thereby interrupting the combustion chain reaction and enhancing the intrinsic safety of the membranes.

Micro‐combustion calorimetry (MCC) is a high‐efficiency technique for investigating the combustion behavior of electrolyte materials, including heat release rate (HRR), total heat release (THR), and carbon dioxide (CO_2_) production [41]. In this study, two electrolyte samples were tested using an MCC (Figure 4f,g). The weight, morphology, and graphitization degree by the MCC effectively reflect the influence of flame‐retardant additives on the char formation process: higher carbon layer generation typically correlates with reduced flammable material and smoke release. As seen in Figure 4f,g, the electrolyte without TEP exhibited minimal carbon residue, even exposing the underlying aluminum foil, whereas the TEP‐added GPE formed a more complete carbon layer (Figure 4f). Its uniform and dense morphology indicates that TEP functions as a flame retardant in both the gas and condensed phase flame retardant. THR and HRR are key indicators for evaluating the fire safety of materials. As shown in Figure 4g, the TFP10v‐GF electrolyte demonstrated lower THR and HRR values compared to FP10v‐GF, indicating that the incorporation of TEP delays electrolyte pyrolysis and reduces peak heat release. Figure S49 further revealed higher CO_2_ generation in FP10v‐GF, while TFP10v‐GF exhibited lower CO_2_ emission. Therefore, these results demonstrate that under high‐temperature pyrolysis conditions, TEP and residual carbon jointly contribute to condensed‐phase flame retardation, effectively interrupting the combustion chain reaction. Meanwhile, the intrinsic thermal stability of AHF‐PMA and the NH• gas generated during pyrolysis further suppress fire propagation. The synergistic effects among these components play a crucial role in comprehensively enhancing battery safety (Figure 4h).

### 2.5 Electrochemical and Mechanical Abuse Resistance of Li|TFP10v‐GF|LFP Pouch Cells

**FIGURE 5:**
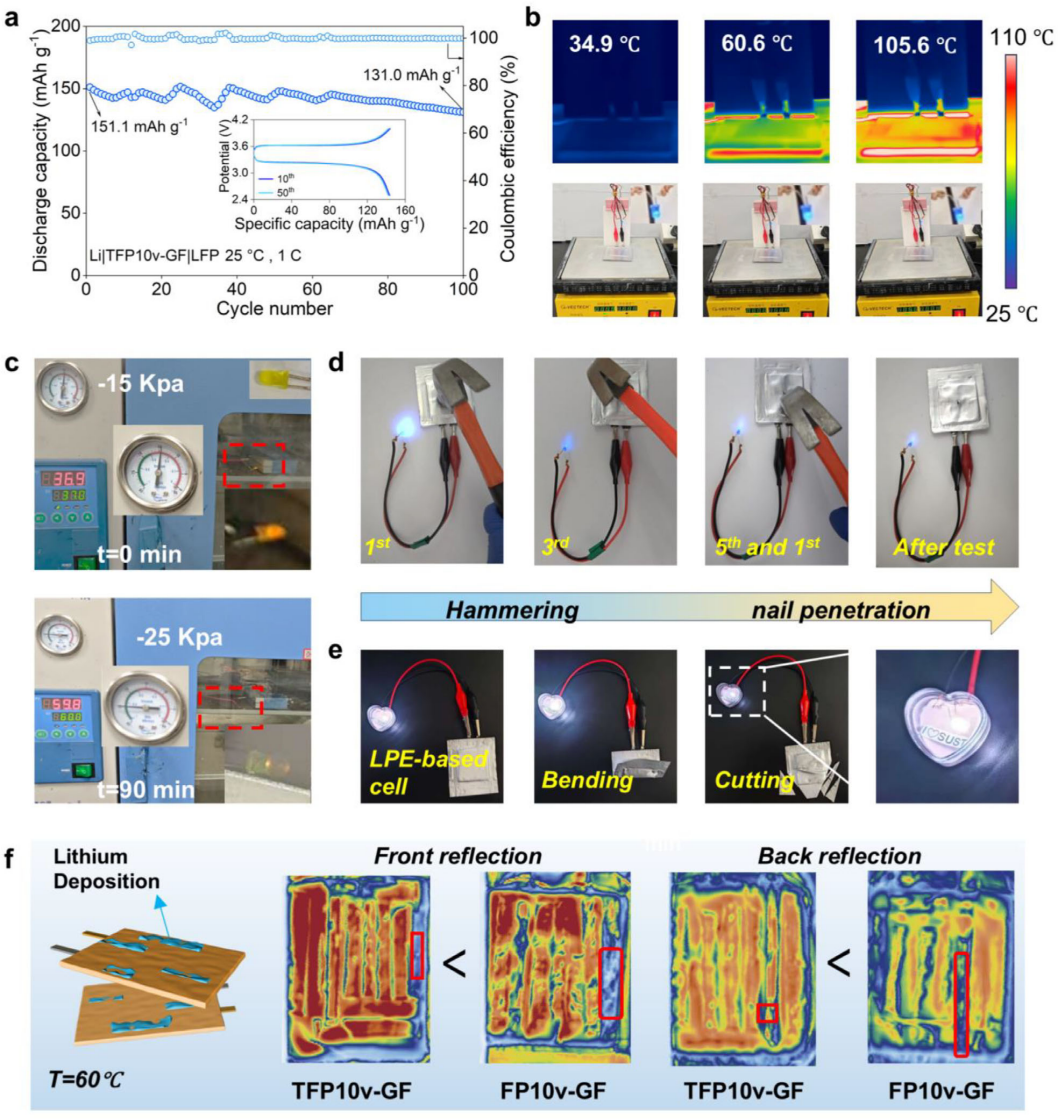
Safety evaluation of Li|TFP10v‐GF|LFP pouch cell. (a) Cycling performance of TFP10v‐GF‐based (Li||LFP) pouch cell at 25°C, and the inset is the corresponding charge‐discharge curves. (b) Thermal stability test. (c) vacuum‐heating test in a vacuum environment of 60°C. (d, e) Digital photographs of pouch cells operating under harsh mechanical abuse, multiple folding, needle puncture tests, and cutting. (f) Static ultrasonic testing of Li|TFP10v‐GF|LFP and Li|FP10v‐GF|LFP pouch cells.

Under abnormal conditions such as impact, overheating, or overcharging, a series of intense exothermic reactions occur at the electrode‐electrolyte interface in LMBs. This leads to rapid heat accumulation within a short period, which can trigger thermal runaway and potentially result in safety incidents such as fires or explosions. Herein, the Li||LFP pouch cell of TFP10v‐GF was assembled to conduct several safety tests. As shown in Figure 5a, the initial charge and discharge coulombic efficiency of the LFP|TFP10v‐GF|Li (N/P = 14.9, E/C = 0.34 g Ah^−1^) was as high as 99.86% after 100 cycles, and the discharge capacity in the 100^th^ cycle was 131.0 mAh g^−1^, which fully proved that TFP10v‐GF had a good electrochemical performance. Furthermore, the highly overlapping charge‐discharge curves after 10 and 50 cycles (inset) further confirm the outstanding capacity retention capability of this battery.

Thermal stability is another crucial performance parameter for practical applications [42]. As shown in Figure 5b and Figure S50, the Li|FP10v‐GF|LFP pouch cell remained stable during the heating process from 25°C to 76.5°C, with the small bulb continuously emitting light. However, when the temperature reached approximately 105°C, the bulb dimmed and then failed. In contrast, the TEP‐added cell (Li|TFP10v‐GF|LFP) maintained normal bulb illumination at 105°C, and the LED‐connected pouch cell exhibited stable lighting without significant swelling or gas release as the hotplate temperature increased from 25°C to 100°C. Additional tests in a vacuum oven simulated high‐temperature and high‐altitude conditions (Figure 5c). Even under extreme conditions of 60°C and ‐25 kPa, the LED continued to emit a weak yet steady glow (Figure S51). These results demonstrate that the TFP10v‐GF‐based battery exhibits excellent safety performance even under harsh conditions, such as high temperatures and low pressures.

Mechanical abuse tests further demonstrated the robustness of the assembled pouch cells. To investigate the safety performance of TFP10v‐GF‐based LMBs under extreme mechanical abuse conditions such as hammering, folding, cutting, and piercing. The results demonstrate exceptional damage resistance and reliability of the battery. As shown in Figure 5d, after repeated hammering tests, although surface dents appeared, the connected LED remained illuminated, indicating excellent mechanical durability. Further systematic abuse tests (Figure 5e) revealed that the pouch cell maintained normal LED lighting even after 180° folding, demonstrating superior flexibility. In the cutting experiment, the battery endured two cutting actions without internal short circuits, fire, or other failures, and continued to supply stable power to the LED. During the nail penetration test, the battery experienced no circuit interruption or failure throughout the piercing and extraction process, with the LED remaining lit continuously (Figure 5e). Even after multiple severe mechanical damages such as bending and cutting, the cycled battery still maintained stable power output, highlighting outstanding mechanical damage resistance, structural integrity, and overall safety reliability.

Non‐destructive ultrasonic testing was conducted to evaluate the internal state of the battery. This technique exploits the propagation of ultrasonic waves, which are highly attenuated in gas relative to solids, to detect Li plating and gas generation at the electrolyte/electrode interface [43]. Li|TFP10v‐GF|LFP and Li|FP10v‐GF|LFP pouch cells were heated at 60°C for 1 h, followed by static ultrasonic testing (Figure 5f). Signal intensity decreases from red (strong) to blue (weak), with blue anomalies indicating possible Li plating or gas formation. Li|TFP10v‐GF|LFP exhibited smaller anomalous regions than Li|FP10v‐GF|LFP in both front and back reflections (Figures S52 and S53), attributed to the superior thermal stability and flame‐retardant properties of TFP10v‐GF. In contrast, larger anomalous areas suggest severe lithium plating, which can induce interfacial dendrite growth, penetration of the separator, and internal short circuits. Such events accelerate capacity degradation and, when combined with mechanical abuse, may trigger thermal runaway, resulting in smoke, fire, or explosion.

### 2.6 Thermal Runaway Evolution and Failure Mitigation in Framework‐Based Electrolytes

**FIGURE 6:**
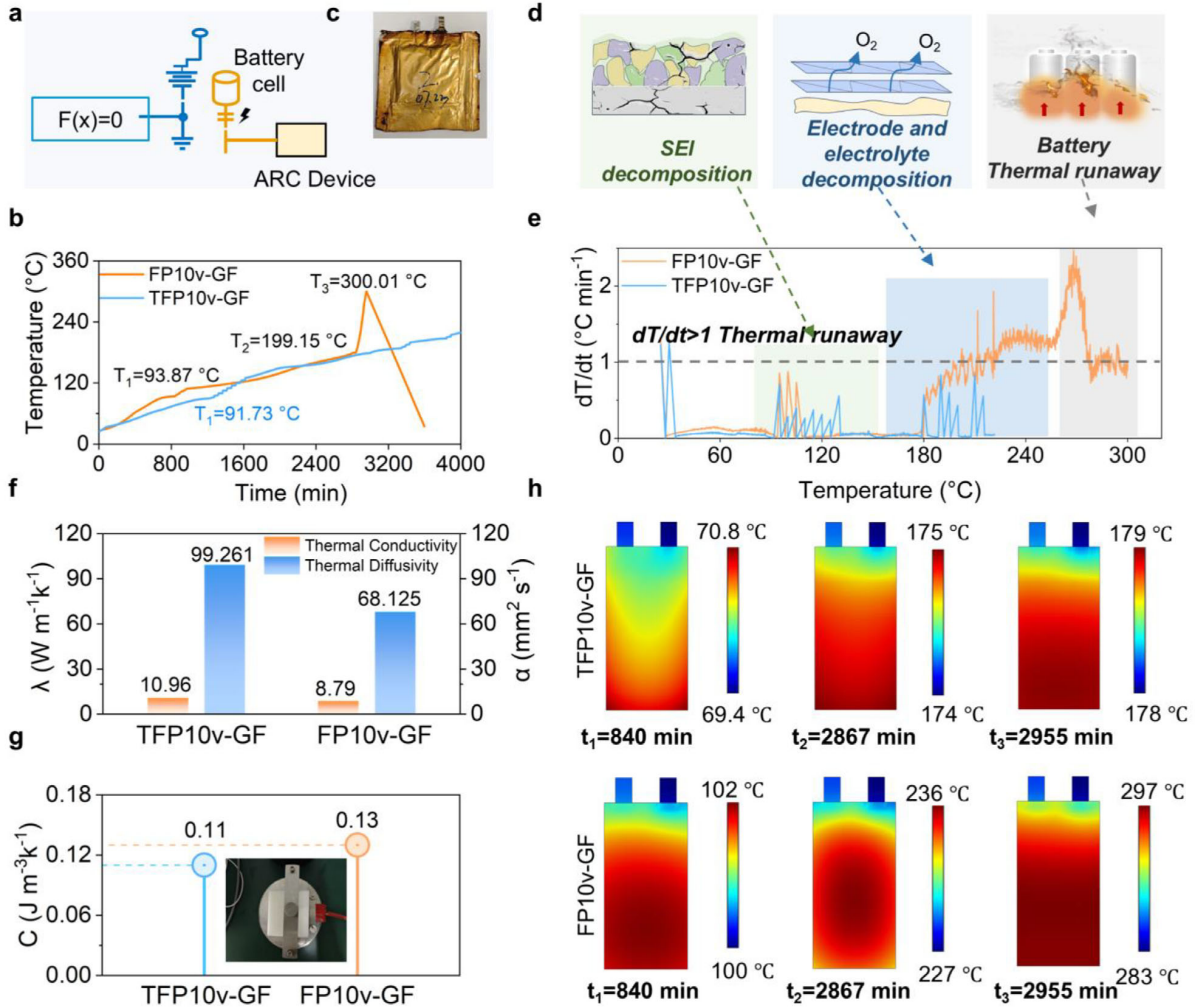
Thermal runaway evaluation of Li|TFP10v‐GF|LFP and Li|FP10v‐GF|LFP pouch cells. (a–e) ARC tests of fully charged Li|TFP10v‐GF|LFP and Li|FP10v‐GF|LFP pouch cells under thermal abuse modes: (a) schematic diagram of ARC tests; (b) temperature versus time curves; (c) optical photographs of the cells after the ARC test; (d) decomposition process of the internal battery components during ARC testing; and (e) corresponding dT/dt curves. (f, g) Comparative diagram of thermal conductivity, thermal diffusivity, and specific heat capacity for pouch cells based on TFP10v‐GF and FP10v‐GF electrolytes. (h) COMSOL simulation of thermal runaway temperature distributions.

Due to the remarkable thermotolerance and superior flame retardance of the TFP10v‐GF, an accelerating rate calorimeter (ARC) was used to study the thermal runaway behavior of TFP10v‐GF‐based and FP10v‐GF‐based pouch cells (Figure 6a–e, Table S5). The thermal safety of fully charged cells was quantitatively assessed using ARC operated in heat‐wait‐search (HWS) protocol (Figure 6a). By defining the onset temperature of exothermic reactions (T_1_, 0.02°C min^−1^) and the thermal runaway trigger temperature (T_2_, 10°C min^−1^), we systematically characterized the thermal runaway behavior [44, 45]. As depicted in Figure 6b, TFP10v‐GF‐based pouch cell exhibits a T_1_ of 93.87°C, which is higher than FP10v‐GF‐based pouch cells (91.73°C), demonstrating its ability to suppress exothermic reactions and delay thermal runaway initiation. Significantly, for the TFP10v‐GF‐based pouch cell, no characteristic thermal runaway temperature point T_3_ was detected during testing. Optical images revealed that the cell remained structurally intact after the entire ARC test (Figure 6c, Figure S54), further indicating an absence of thermal runaway. As shown in Figure 6d,e, the Li|FP10v‐GF|LFP pouch cell reached a thermal runaway temperature (T_3_) of 300.01°C at 2955 min. This behavior is attributed to the combined effects of SEI layer fracture, cathode decomposition, and localized heat accumulation during heating from 25°C to 300°C. In contrast, the Li|TFP10v‐GF|LFP cell exhibited no thermal runaway over the 4000 min test period, confirming that the TFP10v‐GF electrolyte effectively delays thermal runaway and mitigates risks of combustion and explosion.

Thermal conductivity and thermal diffusivity are key thermophysical parameters for evaluating the safety performance of batteries. To this end, we systematically measured the relevant thermal parameters of the battery materials using a hot disk thermal constants analyzer, based on the transient plane source (TPS) method [46]. As shown in Figure 6f, the TFP10v‐GF‐based cell shows enhanced thermal conductivity (*λ* = 10.96 W m^−1^ K^−1^) and thermal diffusivity (*α* = 99.261 mm^2^ s^−1^) relative to FP10v‐GF (*λ* = 8.79 W m^−1^ K^−1^, *α* = 68.125 mm^2^ s^−1^), facilitating faster internal heat dissipation, improved temperature uniformity, reduced thermal runaway risk, and extended lifespan. Moreover, specific heat capacity is a key parameter for evaluating the thermal performance of batteries. To investigate the influence of different material systems, we measured the specific heat capacities of TFP10v‐GF and FP10v‐GF batteries (Figure 6g). The results show that the specific heat capacity of TFP10v‐GF (0.11 J m^−3^ K^−1^) is slightly lower than that of FP10v‐GF (0.13 J m^−3^ K^−1^), which can be attributed to the incorporation of the organophosphate flame retardant TEP with intrinsically low specific heat capacity in TFP10v‐GF. The addition of TEP reduces the overall specific heat capacity of the pouch cell, thereby facilitating faster heat transfer from the internally stored heat to the external environment and, to some extent, enhancing the battery's thermal safety.

Building upon the aforementioned research, we incorporated these parameters into a thermal runaway simulation using COMSOL Multiphysics, obtaining temperature distributions at different time points (Figure 6h). The simulation results indicate that the surface temperature of the Li|TFP10v‐GF|LFP pouch cell remained lower than that of the Li|FP10v‐GF|LFP pouch cell at all times, which can be attributed to its higher thermal diffusivity and thermal conductivity. In contrast, the Li|FP10v‐GF|LFP cell exhibited more pronounced internal heat accumulation, leading to a rapid temperature rise that reached 304.8°C at T_3_ after 2981.9 min (Figures S55 and S56). The minimal 1.6% deviation between the simulated and experimental data further supports the reliability of these findings. In summary, cells incorporating the TFP10v‐GF electrolyte effectively delay thermal runaway, reduce the risk of fire and explosion from local overheating, and extend battery lifespan.

## 3 Conclusion

In conclusion, we have developed a series of self‐assembled azolate hybrid framework‐based solid electrolytes (FPXv‐GF [*X* = 0, 5, 10, 20] and TFP10v‐GF) that simultaneously deliver high ionic conductivity, long‐term cycling stability, and exceptional thermal safety in lithium metal batteries. The well‐defined and continuous framework channels enable accelerated Li^+^ transport, while the synergistic nitrogen‐phosphorus flame‐retardant mechanism effectively suppresses combustion and induces the formation of a thermally insulating, protective carbonaceous layer, thereby impeding heat propagation. The integration of fast ion transport and intrinsic flame retardancy markedly mitigates thermal runaway risks under abusive conditions. Beyond the demonstrated enhancements in electrochemical performance and safety, this work establishes a generalizable design paradigm for multifunctional solid electrolytes, providing new insights and opportunities for the development of next‐generation high‐energy‐density and intrinsically safe lithium metal batteries.

## Author Contributions

**Shun Wang**: writing – review and editing, writing – original draft, formal analysis, data curation. **Qimin Zhu**: validation, conceptualization, writing – original draft. **Yuanyuan Tian**: investigation, data curation. **Peiyu Cui**: visualization, validation. **Qing Ji**: formal analysis, resources, software. **Tian Xie**: methodology, validation, software. **Hong Wang**: conceptualization, validation, project administration. **Dong Zhou**: supervision, data curation, methodology. **Guoxiu Wang**: conceptualization, funding acquisition, project administration, supervision. **Wenhuan Huang**: writing – original draft, funding acquisition, writing – review and editing, project administration, supervision.

## Conflicts of Interest

The authors declare no conflicts of interest.

## Supporting information




**Supporting File 1**: anie73126‐sup‐0004‐SuppMat.docx.

**Supporting File 2**: anie73126‐sup‐0002‐Video.zip.

## Data Availability

The data that support the findings of this study are available from the corresponding author upon reasonable request.
