# Supplementary material for: Flame‐Retardant Quasi‐Solid‐State Electrolytes From Self‐Assembled Azolate Hybrid Frameworks for Highly Safe Lithium Batteries
Source: Angew Chem Int Ed Engl. 2026 Jun 24;65(34):e1279221. doi: 10.1002/anie.1279221 (PMC13480514; doi:10.1002/anie.1279221)
Supplement: Supplementary file 1 [file ANIE-65-e1279221-s001.docx]

Supplementary Information for

**Flame-Retardant Quasi-Solid-State Electrolytes from Self-Assembled Azolate Hybrid Frameworks for Highly Safe Lithium Batteries**

Shun Wang,^#[a,b]^ Qimin Zhu,^#[a,b]^ Yuanyuan Tian,^[a,b]^ Peiyu Cui,^[a,b]^ Qing Ji,^[c]^ Tian Xie,^[a,b]^ Hong Wang, ^[d]^ Dong Zhou,^[e]^ Guoxiu Wang*^[f]^ and Wenhuan Huang*^[a,b,d]^

\# Shun Wang and Qiming Zhu have contributed equally to this work.

[a] Key Laboratory of Chemical Additives for China National Light Industry, College of Chemistry and Chemical Engineering, Shaanxi University of Science and Technology, Xi’an 710021, P. R. China, E-mail: huangwenhuan@sust.edu.cn

[b] Flexible Energy storage and Interfacial Chemistry Key Laboratory of Shaanxi University, College of Chemistry and Chemical Engineering, Shaanxi University of Science and Technology Xi’an 710021, P. R. China., E-mail: huangwenhuan@sust.edu.cn.

[c] Vehicle Energy and Safety Laboratory, Department of Mechanical Engineering, Ningbo University of Technology, Ningbo 315336, P. R. China.

[d] Shanghai Lingfang Energy Co. Ltd., Shanghai 201100, P. R. China.

[e] Tsinghua Shenzhen International Graduate School Tsinghua University Shenzhen 518055.

[f] Centre for Clean Energy Technology, School of Mathematical and Physical Science, Faculty of Science, University of Technology Sydney, Broadway, New South Wales 2007, Australia, E-mail: Guoxiu.Wang@uts.edu.au.

**TABLE OF CONTENTS**

Section 1. Materials Preparation	1-2

Section 2. Methods	3-5

Section 3. Figures and analysis	6-61

Section 4. Supporting tables	62-66

Section 5. Reference	67-68

**Section 1. Materials Preparation**

**1.1 Materials:**

All chemicals used in the synthesis experiments were employed without further purification. Glass fiber (GF) and zinc nitrate hexahydrate (Zn(NO_3_)_2_·6H_2_O, AR grade) were supplied by Tianjin Damao Chemical Reagent Co. Aminotetrazole (5-ATZ), pyromellitic acid (PMA), and tetrapropylammonium hydroxide solution were purchased from Shanghai Aladdin Biochemical Technology Co. Ethanol (C_2_H_6_O); lithium bis(trifluoromethanesulfonyl)imide (LiTFSI, 99%, Aladdin), triethyl phosphate (TEP, 99.7%, Aladdin), poly(ethylene glycol) diacrylate (PEGDA), azobisisobutyronitrile (AIBN, 99%, Aladdin), and commercial liquid electrolyte (fluoroethylene carbonate (FEC) and diethyl carbonate (DEC)); LiFePO_4_ (LFP), conductive carbon black (Super P-Li), polyvinylidene fluoride (PVDF, average Mw ~1000000), and anhydrous N-methyl-2-pyrrolidone (NMP, AR grade) were obtained from Shanghai Aladdin Biochemical Technology Co.

**1.2 Synthesis of AHF-PMA:**

Preparing the zinc salt solution by dissolving zinc nitrate hexahydrate (Zn (NO_3_)_2_.6H_2_O, 0.892 g) in deionized water (H_2_O, 60 mL). The ligand solution by dissolving 5-aminotetrazole (5-ATZ, 0.516 g) and pyromellitic acid (PMA, 0.768 g) in deionized water (H_2_O, 120 mL) and add the diluted tetramethylammonium hydroxide solution drop by drop and use it to adjust the pH (pH≈7). Then uniformly deposit the zinc salt solution (1 mL) on the surface of glass fibers surfaces via a spray-coating process. Subsequently, the ligand solution (2 mL) was spray-coated onto the modified glass fibers, followed by thermal treatment at 170 ℃ to obtain the AHF-PMA composite material. AHF-coated glass fiber films with different loading contents (denoted as 0v-GF, 5v-GF, 10v-GF, and 20v-GF) were prepared by controlling the volume of the sprayed precursor solution.

0v-GF: 0 mL of zinc salt solution and 0 mL of ligand solution.

5v-GF: 5 mL of zinc salt solution and 10 mL of ligand solution.

10v-GF: 10 mL of zinc salt solution and 20 mL of ligand solution.

20v-GF: 20 mL of zinc salt solution and 40 mL of ligand solution.

**1.3 Preparation of electrolytes:**

The FPXv-GF (X=0, 5, 10, 20) and TFP10v-GF were prepared by in-situ polymerization of different monomers with varied proportions. DEC, FEC, TEP and PEGDA were mixed with the volume ratios of 1:1:0:1and 1:1:2:1. The polymer precursor solution, LiTFSI (1M), and AIBN (2 mg mL^-1^) were mixed with vigorous stirring. Then the homogeneous solution with 100 μL was injected into 0v-GF, 5v-GF, 10v-GF and 20v-GF in CR2032-type coin cells, which were adopted as support for the polymer electrolyte and separator between the cathode and anode to prevent the internal short circuit of the battery before the polymerization of the polymer electrolyte. After that, the battery was assembled and transferred to a vacuum oven at 60 ℃ for 1 h to make the complete polymerization of monomer.

**1.4 Preparation of cathode:**

Commercial LiFePO_4_, Super P-Li and PVDF were mixed in NMP with a mass ratio of 7: 2: 1 to obtain the cathode mixture. The slurry was then bladed on Al foil to render uniform coating, which was further dried in a vacuum oven at 100 ℃ for at least 48 h. The high mass loading of the LFP cathode materials was about 7.82 mg cm^-2^. Commercial NCM811, Super P-Li and PVDF were mixed in NMP with a mass ratio of 8:1:1 to obtain the cathode mixture. The slurry was then bladed on Al foil to render uniform coating, which was further dried in a vacuum oven at 100 ℃ for at least 48 h. The obtained electrodes were punched into disks (ϕ=14 mm).

**Section 2. Methods**

**2.1 Characterization**

The powder X-ray diffraction (PXRD) patterns were obtained using a Bruker D2 Phaser X-ray diffractometer with Ni-filtered Cu Kα radiation. The morphology of these samples was observed using a scanning electron microscope (SEM FEI Verios 460) equipped with energy-dispersive X-ray spectroscopy (EDS). Raman spectra of FP10v-GF and FP0v-GF were obtained on Renishaw in via in a frequency range from 700 to 800 cm^-1^. Thermogravimetric analysis (TGA) was performed in the air atmosphere from 30 to 800 °C with a heating rate of 10 °C min^-1^ using TGA-Q500 simultaneous analyzer. The X-ray photoelectron spectroscopy (XPS) analysis was carried out by ESCALAB 250 Xi spectrometer (VG Scientific Co., UK) and all the binding energies obtained in XPS spectra were calibrated using the C 1s peak at 284.6 Ev.

Thermogravimetric analysis-infrared spectrometry (TGA-FTIR, STA 449F3) was chosen to clarify the pyrolysis products of TFP10v-GF and FP0v-GF with a heating rate of 10 °C min^-1^ from 25 ℃ to 600 ℃. Cone calorimeter test (TESTech Instrument, Suzhou) was determined to investigated the fire performance of TFP-10v-GF and its composites according to the standard of ASTM E1354/ISO 5660. The ARC test was conducted in the heat-wait-search mode. The temperature was increased in the step of 5 ℃ followed by a waiting period of 30 min. The characteristic temperatures and times (t_1_ and t_2_) were extracted based on the data analysis. T_1_ is defined as the self-heating temperature of the battery, where self-heating is identified when the battery’s temperature increases rate reaches 0.02 ℃ min^-1^ without external heat. T_2_ refers to the onset temperature of the thermal runaway, which is recorded when the battery’s temperature rise rate (dT/dt) reaches 1 ℃ min^-1^. T_1_ and T_2_ are the self-heating time and thermal runaway onset time of the SMBs, respectively. Transient Plane Source Method (Hot disk TPS2200) for testing thermal conductivity, thermal diffusivity and specific heat capacity.

**2.2 Electrochemical test**

In an argon-filled glove box with H_2_O and O_2_ content below 0.1 ppm, CR2032 coin cells were assembled with polymer electrolytes membranes for various electrochemical tests. The galvanostatic charge-discharge tests were performed on the LAND CT3002A battery test system, the voltage range is 2.5-4.0 V (vs. Li^+^/Li). The tests of cyclic voltammetry (CV) and electrochemical impedance spectroscopy (EIS) are completed on the electrochemical workstation, and the frequency range of the test is 1 kHz~0.01 Hz.

**2.2.1 Ionic conductivity**

Ionic conductivity was determined by EIS after placing the solid electrolyte between two electrodes (stainless steel) contacts in a CR2032 coin cell. CHI660E electrochemical workstation (Shanghai, China) was used for measurements at various temperatures from 25 to 85 °C with a step size of 15 ℃ and frequency ranges from 1 kHz to 0.01 Hz. The bulk resistance of the samples was calculated from the EIS curve.

The ionic conductivity was obtained Via Eq. (1)

$\sigma=\frac{L}{R\times S}$	(1)

where *R* is the resistance value of the bulk electrolyte, *L* is the thickness of electrolyte membrane, and *S* is the area of the stainless-steel electrode.

**2.2.2** **Activation energy (*Ea*)**

The activation energy *Ea* was calculated according to Arrhenius Eq.

$\sigma\left( T \right)={AT}^{-1}e^{-Ea/RT}$	(2)

where *A* was the characteristic constant of the given reaction, *e* is the base of the natural logarithm (2.718), *R* is the gas constant (8.314 J mol^-1^ K^-1^), *T* is the thermodynamic temperature, and *Ea* is the activation energy (kJ mol^-1^).

$\sigma=\sum\frac{Z^{2}FC}{6\pi\eta r_{s}}$·	(3)

where *Z* represents the ionic valence, *C* represents the molar concentration of diffused Li^+^; F denotes Faraday's constant, η denotes the viscosity of electrolyte, and denotes the Stokes radius of solvated Li^+^.

**2.2.3 Electrochemical window**

The electrochemical window of the electrolyte was measured using a liner sweep voltammetry (LSV) mode in the range of 2-6 V at a sweep rate of 10 mV s^-1^. For the LSV test, the electrolyte membrane was sandwiched between the stainless-steel working electrode and the lithium metal foil (as a counter and reference electrode).

**2.2.4 Li^+^ transference number (*t_Li_ ^+^*)**

The electrolyte membrane was sandwiched between two stainless steel plates. The Li^+^ transference number (*t_Li_^+^*) was determined by the chronoamperometry test using symmetric lithium cells at an applied DC voltage of 10 mV. EIS was also performed before and after the DC polarization with the frequency ranging from 10^6^ Hz to 1 Hz.

The *t_Li_^+^* is evaluated by Eq.

$t_{{Li}^{+}}=\frac{I_{s}(\Delta V-I_{0}R_{0})}{I_{0}(\Delta V-I_{s}R_{s})}$	(4)

where *ΔV* is the constant polarization voltage, and the applied voltage is 10 mV, *I_0_* and *R_0_* represent the initial current and resistance, respectively, *I_S_* and *R_S_* are the steady-state current and resistance, respectively.

**2.2.5 Test of Li||Li cells**

The galvanostatic discharge-charge performances of the symmetric cells were evaluated on a LAND battery cycler test system at room temperature. Galvanostatic test of Li||Li symmetric cells were carried out at a current density of 1 mA cm^-2^ and an aerial capacity of 0.2, 0.5, 1, 2 and 5 mAh cm^-2^ at room temperature.

**Section 3. Figures and analysis**

**Fig. S1.** Synthesis and schematic diagram of AHF-PMA.

**Fig. S2.** Schematic diagram of *in-situ* Growth of AHF-PMA.

**Fig. S3.** XRD patterns of the simulation, 5v-GF and AHF-PMA powders.

**Fig. S4.** Thickness of 0v-GF in digital photo.

**Fig. S5.** Optical photographs of 0v-GF, 5v-GF, 10v-GF and 20v-GF.

**Fig. S6.** Flexibility of FP10v-GF in digital photos.

**Fig. S7.** TGA curve of AHF-PMA powders.

**Fig. S8.** Schematic diagram of the *in-situ* thermal polymerization process of electrolyte.

**Fig. S9.** Thickness of FP0v-GF, FP5v-GF, FP10v-GF and FP20v-GF in digital photos.

**Fig. S10.** SEM images of (a) 0v-GF and (b) FP0v-GF.

**Fig. S11.** SEM images of (a) 5v-GF and (b) FP5v-GF.

**Fig. S12.** SEM images of (a) 10v-GF and (b) FP10v-GF.

**Fig. S13.** SEM images of (a) 20v-GF and (b) FP20v-GF.

**Fig. S14.** SEM images of the 0v-GF membrane and the corresponding O and

Si elemental mappings.

**Fig. S15.** SEM images of the FP0v-GF membrane and the corresponding C, O, Si and F elemental mappings.

**Fig. S16.** SEM images of the 5v-GF membrane and the corresponding C, N, O, Zn and Si elemental mappings.

**Fig. S17.** SEM images of the FP5v-GF membrane and the corresponding C, N, O, Zn, Si and F elemental mappings.

**Fig. S18.** SEM images of the 10v-GF membrane and the corresponding C, N, O, Zn and Si elemental mappings.

**Fig. S19.** SEM images of the FP10v-GF membrane and the corresponding C, N, O, Zn, Si and F elemental mappings.

**Fig. S20.** SEM images of the 20v-GF membrane and the corresponding C, N, O, Zn and Si elemental mappings.

**Fig. S21.** SEM images of the FP20v-GF membrane and the corresponding C, N, O, Zn, Si and F elemental mappings.

**Fig S22.** The electrostatic potential of AHF-PMA.

**Fig S23.** Binding energy of Li^+^ and TFSI^-^ on AHF-PMA.

**Fig. S24.** The EIS spectra of the symmetric SS||SS batteries (a) FP0v-GF, (b) FP5v-GF, (c)FP10v-GF and (d) FP20v-GF membranes for calculating the Li ionic conductivity at a temperature range of 25-85 ℃.

**Fig. S25.** Lithium ions conductivity of FP0v-GF, FP5v-GF, FP10v-GF and FP20v-GF as a function of temperature.

**Fig. S26.** Chronoamperometry curves and AC impedance spectroscopy of (a) FP0v-GF, (b) FP5v-GF, (c)FP10v-GF and (d) FP20v-GF.

**Fig. S27.** Equivalent Circuit Model for Fitting AC Impedance Spectroscopy (EIS) Data of Li||Li Symmetric Cells

**Fig. S28.** Tafel plot for Li plating/stripping of the Li||Li symmetric batteries.

**Fig. S29.** Rate performance of Li symmetric cell with FP10v-GF membrane.

**Fig.30.** XRD patterns of Simulated, AHF-PMA powder, FP10v-GF membrane, and cycled FP10v-GF’ membrane after 100 h operation in Li symmetric cell (Li||FP10v-GF|Li).

**Fig. S31.** The voltage-capacity curves and coulombic efficiency of Li||Cu cells.

**Fig. S32.** LSV curves of FP0v-GF, FP5v-GF, FP10v-GF and FP20v-GF.

**Fig. S33.** Calculated HOMO/LUMO energy levels of PEGDA, DEC, FEC, AHF-PMA and LiTFSI.

**Fig. S34.** XPS spectra of sodium anode after 50 cycles with Li|FP10v-GF|Li symmetric battery.

**Fig. S35.** XPS spectra of sodium anode after 50 cycles with Li|FP0v-GF|Li symmetric battery.

**Fig. S36.** CV curves of the Li|FP0v-GF|LFP, Li|FP5v-GF|LFP, Li|FP10v-GF|LFP and Li|FP20v-GF|LFP cells at 0.1 mV s^-1^.

**Fig. S37.** Corresponding galvanostatic charge discharge curves of (a) Li|FP0v-GF|LFP, (b) Li|FP5v-GF|LFP and (c) Li|FP20v-GF|LFP cells cycling from 0.1 to 2 C.

**Fig. S38.** Cycling performance of the Li|FP10v-GF|LFP battery at 2 C for 400 cycles.

**Fig. S39.** Cycling performance of the Li|FP10v-GF|NCM811 battery at 0.1 C for 10 cycles.

**Fig. S40.** Cycling performance of the Li|FP10v-GF|NCM811 battery at 1 C for 30 cycles.

**Fig. S41.** SEM images of the TFP10v-GF membrane and the corresponding C, N, O, Zn, Si, F and P elemental mappings.

**Fig. S42.** a) ElS measurement of TFP10v-GF. b) Ionic conductivities of TFP10v-GF at diﬀerent temperatures. c) Activation energies of TFP10v-GF.

**Fig. S43.** Chronoamperometry profile of Li|TFP10v-GF|Li cells under 10 mV polarization voltage.

**Fig. S44.** Calculated HOMO/LUMO energy levels of DEC, FEC, TEP and LiTFSI.

**Fig. S45.** P 2P in XPS spectra of cycled Lithium metal interface FP10v-GF and TFP10v-GF electrolyte surface.

**Fig. S46.** Infrared thermography of TFP10v-GF at 37.8-300.5 °C.

**Fig. S47.** Flammability tests of GF, TFP10v-GF and FP10v-GF.

**Fig. S48.** 3D TGA‐FTIR heatmap of (a) FP10v-GF and (b) TFP10v-GF membranes.

**Fig. S49.** The micro-combustion calorimeter (MCC) test of TFP10v-GF and FP10v-GF membranes from 500 °C: carbon dioxide (CO_2_) release curves.

**Fig. S50.** Thermal stability test of LFP|FP10v-GF|Li pouch cell.

**Fig. S51.** Vacuum-heating test in a vacuum environment of Li|TFP10v-GF|LFP pouch cell.

**Fig. S52.** Static ultrasonic testing of Li|TFP10v-GF|LFP pouch cell.

**Fig. S53.** Static ultrasonic testing of Li|FP10v-GF|LFP pouch cell.

**Fig. S54.** Optical photograph of the post-ARC-test Li|TFP10v-GF|LFP pouch cell.

**Fig. S55.** Temperature vs. time curves of Li|TFP10v-GF|LFP pouch cell in ARC test with heat-wait-seek (HWS).

**Fig. S56.** Temperature vs. time curves of Li|FP10v-GF|LFP pouch cell in ARC test with heat-wait-seek (HWS).

**Section 4. Supporting Tables**

**Table S1.** Li^+^ ionic conductivity of FP0v-GF, FP5v-GF, FP10v-GF and FP20v-GF membranes.

| **Temperature (℃)** | ***σ* (S cm^-1^)** | | | | |
| --- | --- | --- | --- | --- | --- |
|  | **FP0v-GF** | **FP5v-GF** | **FP10v-GF** | **FP20v-GF** | **TFP10v-GF** |
| 25 | 6.97×10^-4^ | 1.0×10^-3^ | 1.44×10^-3^ | 3.78×10^-4^ | 1.36×10^-3^ |
| 35 | 7.71×10^-4^ | 1.10×10^-3^ | 1.61×10^-3^ | 4.42×10^-4^ | 1.42×10^-3^ |
| 45 | 9.54×10^-4^ | 1.34×10^-3^ | 1.91×10^-3^ | 5.27×10^-4^ | 1.62×10^-3^ |
| 55 | 1.09×10^-3^ | 1.51×10^-3^ | 2.08×10^-3^ | 6.16×10^-4^ | 1.85×10^-3^ |
| 65 | 1.24×10^-3^ | 1.72×10^-3^ | 2.34×10^-3^ | 7.07×10^-4^ | 2.11×10^-3^ |
| 75 | 1.38×10^-3^ | 1.89×10^-3^ | 2.67×10^-3^ | 7.96×10^-4^ | 2.42×10^-3^ |
| 85 | 1.54×10^-3^ | 2.08×10^-3^ | 2.97×10^-3^ | 8.74×10^-4^ | 2.60×10^-3^ |

**Table S2.** Li^+^ transference number (*t_Li_^+^*) and Activation energy (*Ea*) of FP0v-GF, FP5v-GF, FP10v-GF FP20v-GF and TFP10v-GF membranes

|  | **FP0v-GF** | **FP5v-GF** | **FP10v-GF** | **FP20v-GF** | **TFP10v-GF** |
| --- | --- | --- | --- | --- | --- |
| ***t_Li_^+^*** | 0.43 | 0.49 | 0.63 | 0.18 | 0.72 |
| ***Ea* (eV)** | 0.116 | 0.108 | 0.103 | 0.122 | 0.105 |

**Table S3.** Comparison of cycle properties of QSSLMBs with previously reported gel polymer electrolytes.

| **Component** | ***t_Li_^+^*** | ***σ* (S cm^-1^)** | ***Ea* (eV)** | **Publish Year** | **Ref.** |
| --- | --- | --- | --- | --- | --- |
| **FRSPE** | 0.42 | 1.81×10^-4^ (25 ℃) | 0.34 | 2024 | Ref. [1] |
| **Janus@GPE** | 0.57 | 1.00 ×10^-3^ (25 ℃) | 0.49 | 2024 | Ref. [2] |
| **LiNO_3_-PPT@PI** | 0.62 | 7.57×10^−4^ (25 ℃) | _ | 2024 | Ref. [3] |
| **GEL@F** | 0.56 | 1.063×10^-3^ (25 ℃) | _ | 2024 | Ref. [4] |
| **CIL-MOF/PVDF** | 0.40 | 1.14×10^-3^ (25 ℃) | 0.126 | 2025 | Ref. [5] |
| **2P(AT-MBA6F)** | 0.50 | 1.40×10^-3^ (25 ℃) | 0.20 | 2025 | Ref. [6] |
| **FL_7_M_3_@CSPE_SO_** | 0.52 (30 ℃) | 4.86×10^-4^ (30 ℃) | _ | 2025 | Ref. [7] |
| **T-SPE** | 0.56 | 9.00 ×10^-4^ (25 ℃) | 0.18 | 2024 | Ref. [8] |
| **LDR** | 0.46 | 1.35 ×10^-4^ (25 ℃) | 0.279 | 2024 | Ref. [9] |
| **PAN fiber-PEGDA** | 0.42 | 8.80×10^-4^ (25 ℃) | 0.35 | 2024 | Ref. [10] |
| **TFP10v-GF** | 0.72 | 1.36×10^-3^ (25 ℃) | 0.105 | 2026 | **This work** |
| **FP10v-GF** | 0.63 | 1.44 ×10^-3^ (25 ℃) | 0.103 | 2026 | **This work** |

**Table S4.** The comparison of performances of different QSSLMBs.

| **Component** | **Cycle performance (mAh g^-1^)** | **Rate performance (mAh g^-1^)** | **Ref.** |
| --- | --- | --- | --- |
| **SGPE** | 110.4 (0.5 C, RT), 96.1 (1 C, RT), 72.1 (2 C, RT) | 153.8 (0.1 C, RT), 147.9 (0.2 C, RT), 136.3 (0.5 C, RT), 124 (1 C, RT), 107.7 (2 C, RT） | Ref. [11] |
| **PMLSE** | 127.7 (0.2 C, RT), 116.5 (1 C, RT) | 142.8 (0.1 C, RT), 138.9 (0.2 C, RT), 136.3 (0.5 C, RT), 127.5 (1 C, RT) | Ref. [12] |
| **HECE** | 137.7 (0.5C, RT) | 158.9 (0.1 C, RT), 154.0 (0.2 C, RT), 140.9 (0.5 C, RT), 107.1 (1 C, RT) | Ref. [13] |
| **PLZM-7** | 145.5 (0.1C, 60℃), 112.5 (0.1C, 30℃) | 159.48 (0.1 C, 60℃), 148.03 (0.2 C, 60℃), 114.27 (0.5 C, 60℃), 70.35 (1 C, 60℃) | Ref. [14] |
| **PolyIL-based binder** | 122.9 (0.5 C, RT) | 157.5 (0.1 C, RT), 152.2 (0.2 C, RT), 137.5 (0.5 C, RT), 122.5 (1 C, RT), 105.5 (2 C, RT) | Ref. [15] |
| **PCLA QPE** | 126 (1 C，RT) | 154.21 (0.1 C), 142.04 (0.2 C), 130.22 (0.5 C), 119.24 (1 C), 105.23 (2 C), 82.8 (5 C) | Ref. [16] |
| **PTHLZ** | 164.4 (0.1 C, RT) | 164.4 (0.1 C, RT). 156.4 (0.2 C，RT), 60.6 (5 C,RT) | Ref. [17] |
| **ELPO** | 97.1 (0.2 C, RT) | 114.8 (3C, RT) | Ref. [18] |
| **Polymer** | 86 (5C, RT) | 113 (0.2 C，RT), 114 (0.5 C，RT), 113 (1 C，RT), 110 (5 C，RT), 103 (10 C，RT), 95(15 C，RT) | Ref. [19] |
| **FP10v-GF** | 148.53 (0.1 C, RT), 140.30 (0.1C, high loading), 132.03 (1 C, RT), 116.58 (2 C), 146.27 (1 C, 60℃) | 146.6 (0.1 C, RT), 144.2 (0.2 C, RT),139.5 (0.5 C, RT), 131.4 (1 C, RT), 116.9 (2C, RT） | **This work** |

**Table S5.** Time-temperature parameters during thermal runaway in different pouch cells

| **Component** | **T_1_ (℃)** | **T_2_ (℃)** | **T_3_ (℃)** | **Ref.** |
| --- | --- | --- | --- | --- |
| **PFGPE** | 30.0 ℃ | 219.5 ℃ | 356.3 ℃ | Ref. [20] |
| **QSEs (50%)** | 25.0 ℃ | 127.8 ℃ | 345.0 ℃ | Ref. [21] |
| **GPE** | 25.0 ℃ | 155.38 ℃ | 505.0 ℃ | Ref. [22] |
| **FP10v-GF** | 93.87 ℃ | 199.15 ℃ | 300.01 ℃ | This work |
| **TFP10v-GF** | 91.73 ℃ | _ | _ | This work |

**Section 6. References**

1.	Z. Li, S. Zhu, S. Gao, Y. He, H. Ding, D. Yang, H. Yang, P. F. Cao, *Adv. Funct. Mater*., 2024, 34, 2409836.

2.	L. Wang, S. Xu, Z. Song, W. Jiang, S. Zhang, X. Jian, F. Hu, *InfoMat* **2024**, 6, e12551.

3.	X. Wang, L. Xu, M. Li, Y. Hu, N. Wang, Y. Meng, K. Yang, K. Deng, Energy Storage Materials. *Energy Storage Materials* **2024**, 73, 103778.

4.	K. Zeng, Q. Liu, H. Ma, G. Zhao, Q. An, C. Zhang, Y. Yang, M. Sun, Q. Xu, L. Duan, H. Guo, *Energy Storage Materials* **2024**, 70, 103564.

5.	M. Gao, D. Zhou, B. Wen, S. Zhu, J. Ni, *Adv. Funct. Mater.* **2025**, 35, 2500727.

*6.*	G. Ye, X. Hong, M. He, J. Song, L. Zhu, C. Zheng, Y. Ma, Y. An, K. Shen, W. Shi, Y. Jia, M. B. Shafqat, P. Gao, D. Xia, F. Chen, Q. Pang, *Adv. Mater.* **2025**, 37, 2417829.

7.	Y. Jiang, L. Liu, Y. Liu, J. Guan, H. Wang, M. Zhang, L. Chen, Y. Cao, R. Li, Y. Zhou, Q. Zeng, Z. Li, W. Liu, X. Li, L. Zhang, *Adv. Funct. Mater.* **2025**, 35, 2502613.

8.	J. Han, M. J. Lee, J. H. Min, K. H. Kim, K. Lee, S. H. Kwon, J. Park, K. Ryu, H. Seong, H. Kang, E. Lee, S. W. Lee, B. J. Kim. *Adv. Funct. Mater.* **2024**, 34, 2310801.

9.	R. H. Wang, W. Wang, Y. Z. Zhang, W. Hu, L. Yue, J. H. Ni, W. Q. Zhang, G. Pei, S. Yang, L. F. Chen. *Angew. Chem. Int. Ed.* **2024**, 64, e202417605.

10.	Y. Zhang, J. Yu, H. Shi, S. Wang, Y. Lv, Y. Zhang, Q. Yuan, J. Liang, T. Gao, R. Wei, X. Chen, L. Wang, Y. Yu, W. Liu. *Adv. Funct. Mater.* **2025**, 35, 2421054.

11.	Q. Sun, Z. Gong, T. Zhang, J. Li, X. Zhu, R. Zhu, L. Wang, L. Ma, X. Li, M. Yuan, Z. Zhang, L. Zhang, Z. Qian, L. Yin, R. Ahuja, C. Wang. *Nano-Micro Letters* **2024**, 17, 18.

12.	J. Sun, X. Yao, Y. Li, Q. Zhang, C. Hou, Q. Shi, H. Wang, *Adv. Energy Mater.* **2020**, 10, 2000709.

13.	S. Yuan, Z. Cao, Z. Zhao, S. Lv, Y. Zhai, Z. Ji, R. Li, Y. Wang, X. Fu. *Adv. Funct. Mater.* **2025**, 35, 2503663.

14.	J. Li, R. Yang, A. Jiang, Q. Zhang, X. Dong, H. Shang, Y. Yan, Y. Xu, J.-H. Ahn. *Chemical Engineering Journal* **2025**, 514, 162820.

15.	J. Li, H. Hu, J. Zhu, X. Ma, Y. Hu, H. Zhang, F. Liu, S. Zhang, X. Ji. *Adv. Mater.* **2025**, 37, 2501659.

16.	Y. Liu, C. Ye, Y. Chen, Y. Cheng, Y. Ding, S. Tang. *Angew. Chem. Int. Ed.* **2025**, 64, e202516098.

17. H. Liu, D. Li, Y. Yang, Y. Lan, X. Zhao, T. Zhong, T. Hu, S. Luo, M. Guan, Y. Li. *Adv. Energy Mater.,* **2025**, 15, 2501350.

18. J. Zhu, B. Ma, C. Zhong, Z. Wang, C. Tian, S. Weng, S. Li, M. Cao, Z. Wang, Y. Li, H. Li, T. Cheng, X. Wang. *Adv. Energy Mater.,* **2025**, 16, e04413.

19. Z. Li, J. Chen, Q. Fang, J. Fu, Y. Ren, X. Wang, X. Guo. *Angew. Chem. Int. Ed.,* **2025**, 64, e202505509.

20.	B. Yang, Y. Pan, T. Li, A. Hu, K. Li, B. Li, L. Yang, J. Long. *Energy Storage Materials* **2024**, 65, 103124.

21.	W. Li, H. Li, J. Liu, S. Lin, Q. Chen, W. Ji, Z. He, P. Zhang, J. Zhao. *Energy & Environmental Science* **2023**, 16, 5444-5453.

22.	X. Cheng, C. Lu, X. Gong, C. Li, J. Wang, J. Qu, Y. Zhang, T. Song, Y. Zhang, H. Jiang, C. Wang, Y. Long, Y. Cao, Y. Wang, W. Li, H. Peng, B. Wang, Angew. Chem. Int. Ed. **2025**, 64, e202423419.
